# Effect of resistance vs. aerobic exercise in pre-diabetes: an RCT

**DOI:** 10.1186/s13063-023-07116-3

**Published:** 2023-02-14

**Authors:** Xijuan Luo, Zhengzhen Wang, Bowen Li, Xianbo Zhang, Xin Li

**Affiliations:** 1 Department of Sports, Sun Yat-sen University, Guangzhou, 510275 China; 2 Beijing Sport University, Beijing, 100084 China; 3 School of Sports and Health, Nanjing Sport Institute, Nanjing, 210014 China; 4 Department of Endocrinology, Beijing Hospital, National Center of Gerontology, Institute of Geriatric Medicine, Chinese Academy of Medical Sciences, Beijing, 100730 China; 5 Center of Academic Journals, Chengdu Sport University, Chengdu, 610041 China

**Keywords:** Aerobic exercise, Exercise prescription, IGR, IR, Pre-diabetes, Resistance exercise

## Abstract

**Background:**

This study aimed to assess the different impacts of aerobic and resistance exercise intervention on pre-diabetes and its possible influencing factor (obesity) to identify which exercise intervention mode was better for pre-diabetes to control their blood glucose levels.

**Methods:**

Single-blind randomized controlled trial. Participants were recruited from Southwest Hospital between February 2016 and May 2017 and randomly divided into three groups using stratified randomization: aerobic exercise (A), resistance exercise (R), and control (C). The effects of each group were analyzed, and the relationship with obesity was investigated following a 12-week intervention.

**Results:**

Eighty participants were enrolled (9 were lost, and 1 was excluded). Finally, 26 participants were included in group A, 23 in group R, and 21 in group C. In groups A and R, FPG, OGTT 2-h PG, and HOMA2-IR decreased by 6.17% (*P* = 0.001) and 4.81% (*P* = 0.019), 20.39% (*P* < 0.001) and 16.50% (*P* < 0.001), and 8.34% (*P* = 0.026) and 18.31% (*P* = 0.001, superior to that in group A), respectively (all *P* < 0.001 compared with group C, with no significant differences between groups A and R). The ratio of reversal to euglycemia was 69.2% (*P* = 0.003 compared with group C) in group A and 43.5% (*P* = 0.213 compared with group C) in group R. The decreased ratio of GSP in group R was greater (65.2%, *P* = 0.008 compared with group C) compared with group A (38.5%, *P* = 0.355 compared with group C). Decreases in the parameters BMI (3.1 ± 3.2% *P* < 0.001, moderately positive correlation with the decreased FPG level, *r* = 0.498, *P* = 0.010, two-tailed) and waist circumference (3.1 ± 2.7% *P* < 0.001) were noted in group A, but no significant correlations were noted between other indicators in group R.

**Conclusions:**

Both resistance and aerobic exercise can control and reverse IGR. Compared with aerobic exercise, resistance exercise may be superior in terms of GSP and IR improvement. Aerobic exercise decreases blood glucose levels through weight loss. However, the effect of resistance exercise might not be mediated via weight loss and obesity control.

**Trial registration:**

Chinese Clinical Trial Registry ChiCTR2000038304. Registered on September 17, 2020.

## Introduction

Changes in lifestyle have rapidly increased the levels of prevalence associated with diabetes mellitus (DM) and pre-diabetes [or impaired glucose regulation, IGR, which includes impaired fasting glucose (IFG) and/or impaired glucose tolerance (IGT)] worldwide. This severely affects the quality of life and is a life-threatening development. The International Diabetes Federation (IDF) reported in 2021 that the global prevalence of diabetes reached 10.5% (537 million adults), which was a rise of 16% (74 million) since the previous IDF estimates in 2019. The prevalence is predicted to increase to 11.3% (643 million) by 2030 and to 12.2% (783 million) by 2045. Furthermore, 10.6% (541 million) of adults worldwide have IGT, placing them at high risk of developing type 2 diabetes [1]. In China, the prevalence of DM detected using the World Health Organization criteria was 11.2%, and the prevalence of DM and pre-diabetes detected using the American Diabetes Association criteria was 12.8% and 35.2%, respectively, among adults [2]. The prevalence of IGR in some reports even reached 50.1% (493.4 million), representing a huge reservoir of the DM population [3]. IGR is an intermediate stage of development between normal and DM states. It can persist, return to a normal state, or progress to DM. Approximately 60% of patients with type 2 DM (T2DM) manifest IGR 5 years prior to the onset of this condition [4]. IGR is reversible, and aerobic exercise combined with health education can decrease the prevalence of DM by 58% [5]. Therefore, the control and reversal of IGR and the prevention of T2DM are challenging.

Exercise is extremely important to prevent DM in individuals with IGR. Although the benefit of aerobic exercise in IGR has been confirmed [6, 7], the impact of resistance exercise on IGR remains unclear. It seems that resistance exercise is beneficial for glycemic control in people with IGR and is different from aerobic exercise [8, 9]. Therefore, the Intercepting Diabetes Project was undertaken in China. The present study was part of this project. The different effects of aerobic and resistance exercise on IGR were analyzed, and the relationship with obesity was investigated to identify the possible influencing factors so as to explore which exercise intervention mode was better for individuals with IGR and for the control of their blood glucose levels. The findings might provide a theoretical and practical basis for DM prevention and IGR management.

## Methods

### Participant recruitment and screening

**Fig. 1:**
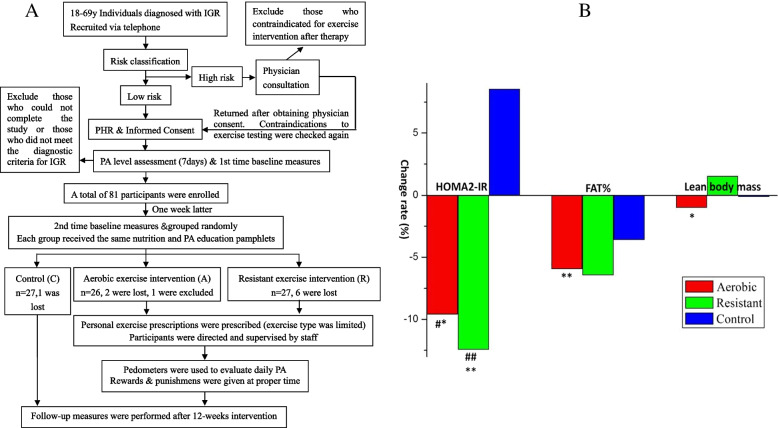
**A** Study protocol route chat. First time baseline measures included fasting plasma glucose (FPG), 2hPG following OGTT, fasting insulin (FINS), glycosylated serum protein (GSP), cardiorespiratory fitness (CRF) and 7-day PA assessment. Second time baseline measures included height, weight, BMI, waist circumference, muscular strength, and body composition determined using BIA. **B** Ratio of change of HOMA2-IR, FAT%, Lean body mass in 3 groups with IGR. *P<0.05, **P<0.01, compared with pre-intervention within group; ^#^*P*<0.05, ^##^*P*<0.01, compared with group C

The study protocol is outlined in Fig. 1A. Individuals diagnosed with IGR, aged 18–69 years, were screened during the physical examination by physicians of the Southwest Hospital Health Management Center from February 1, 2016, to May 1, 2017. The participants were recruited via telephone and referred to our exercise intervention center in the Southwest Hospital Health Management Center to undergo the oral glucose tolerance test (OGTT) again.

Based on the pre-participation health screening of the American College of Sports Medicine (ACSM), the screening questionnaire and 2014 Physical Activity Readiness Questionnaire (2014 PAR-Q+) were used to classify the exercise risk and recommend individuals at exercise high risk (any answer in 2014 PAR-Q+ was “yes”) for physician consultation [10]. The patients returned to our exercise intervention center after obtaining physician consent. The participants’ basic information was recorded, including sex, age, contact, time point of initial abnormal blood glucose detection, and drug use.

All participants provided written informed consent, and their personal health records were established. Ethics approval was granted by the ethics committee of Beijing Sport University (Beijing, China). The Institutional Review Board Approval Number was 2016012, and the registration number was ChiCTR2000038304.

The following inclusion criteria were used: (1) individuals aged between 18 and 69 years, with OGTT meeting Chinese IGR diagnostic criteria (6.1 mmol/L ≤ FPG ≤ 7.0 mmol/L and/or 7.8 mmol/L ≤ OGTT 2-h PG ≤ 11.1 mmol/L) and voluntarily participating in exercise; (2) individuals classified as exercise low-risk (answers in 2014 PAR-Q+ were all “no”) and those classified as exercise high risk whose conditions were controlled following therapy and who were approved by physicians to participate in exercise intervention in the absence of contraindications [2]. The following exclusion criteria were used: (1) exercise high-risk individuals contraindicated for exercise intervention following therapy; (2) individuals who could not complete the study (for example, due to migration or long-term business); (3) those whose OGTT outcome did not meet the diagnostic criteria for IGR in the baseline test; and (4) those who received medication for hyperglycemia or hyperlipidemia.

### Grouping

This study was a single-blind randomized controlled trial. Stratified randomization was used for grouping. After stratified by age bracket (every 20 years was an age bracket) and sex, a computer random number generator was used to divide the participants randomly into three groups as follows: aerobic exercise intervention (A), resistance exercise intervention (R), and control (C). Intervention and statistics staff were aware of the groupings, but none of the participants was aware of the presence of the other groups (single-blind experimental design). The sample size estimation formula for the comparison of multiple samples was used to estimate the sample size (*α* = 0.05, *β* = 0.1). Based on the blood glucose value of the preliminary experiment and previous related studies (the level in group A was 5.58 ± 0.61 mmol/L prior to intervention and 5.32 ± 0.59 mmol/L following intervention; the level in group R was 8.14 ± 1.57 mmol/L prior to intervention and 7.5 ± 1.63 mmol/L following intervention, and the level in group C was 5.94 ± 0.54 mmol/L prior to intervention and 6.11 ± 0.46 mmol/L following intervention), the minimum sample size of each group was calculated as 10 and the expected loss ratio was 30%. Therefore, the initial sample size was at least 39, with an average of 13 individuals in each group.

### Intervention plan

All three groups received the same nutrition and physical activity (PA) education pamphlets. The contents of the pamphlets included IGR-related health information and lifestyle interventions, and suggestions for diet and exercise to prevent diabetes with appropriate use of common sense during exercise and precautions.

Prior to intervention, anaerobic and resistance exercise prescription software for IGR was developed according to the ACSM’s exercise testing and prescription (10th edition) guidelines and other previous studies [7, 11, 12]. Based on the baseline measurements, personal exercise prescriptions were given to the individuals in the two exercise intervention groups. The principles for the prescriptions were as follows: (1) In group A, the exercise type included walking or running, combined with aerobic gymnastics and swimming as required (notably for those who exhibited limited joint movement and could not walk or run sufficiently). The exercise was performed 3 days a week and lasted 50 min/day of continued training. The exercise intensity was 40–49% V̇O_2_R within the first 1–4 weeks, followed by 50–59% V̇O_2_R within 5–12 weeks. (2) In group R, the exercise type included elastic band progressive resistance training, 3 days a week, with at least 48 h separating the exercise training sessions. Furthermore, 5–10 movements were used for the major muscle group (i.e., chest, shoulders, upper and lower back, abdomen, hips, and legs) training. Each muscle group was trained with two to three sets with a rest interval of 2–10 min, and 8–16 repetitions per set, which also accounted for 50 min/day of exercise in total. The exercise intensity was 14 to ≥16 repetition maximum (RM) in training stage 1, 12–16 RM in training stage 2, and 8–12 RM in training stage 3. Resistance exercise was performed at a moderate speed (controlled by music and completing a single movement in ~2 s) [11]. The exercise load was adjusted according to individual capacity. In case the participant could easily tolerate 16 repetitions per set under a given load, he/she proceeded to the next stage at a higher load following a re-evaluation of his/her muscular strength. Warm-up and cool-down exercises were performed prior to and following aerobic or resistance exercises.

### Implementation, supervision, and management

Prior to the experiment, the staff was trained according to the experimental operation manual and held responsible for a limited number of participants. The participants in the two exercise groups were all directed and supervised by relevant staff face to face according to their personal exercise prescriptions. The exercise intensity and volume were monitored using heart rate (HR), rating of perceived exertion (RPE), and pedometers. The safety of exercise intervention was ensured through pre-exercise health screening and clinical exercise testing. Necessary medical supervision was carried out during exercise testing and exercise intervention to monitor and record the occurrence of sports injuries, diseases, accidents (cardiovascular events, hypoglycemia, and trauma), and other adverse effects. At the test and intervention sites, appropriate exercise and first-aid equipment were prepared, the knowledge and skills to deal with adverse events were provided to staff, and health education of also individuals was conducted to improve the cognition and prevention awareness of adverse events so as to minimize the incidence of adverse events. Once an adverse event occurred during the experiment, the experimental operator operated according to the workflow of the pre-established emergency plan and recorded the specific situation. The doctor or medical institution identified the adverse event. Fortunately, no adverse events occurred during the trial.

All data, including drug use, injury, disease, accident, pedometer data, strict attendance, outcomes, and so on, were recorded and collected by these special staff according to the experimental operation manual and kept confidential to ensure the privacy of participants. Then, the original data were entered, sorted out, checked, and kept by specialized data management personnel to ensure the accuracy and safety of the data. The absentee was educated and encouraged to be punctual. At the end of the 12th weekend, the attendance was reviewed. Participants whose attendance was less than 75% (a delay exceeding 30 min was considered as absence) were excluded, and the follow-up tests were also canceled. The participants with optimal attendance were encouraged verbally or through incentives.

### Outcomes

The main outcomes included the following: fasting blood glucose (FPG), 2-h glucose following OGTT, fasting insulin (FINS), glycosylated serum protein (GSP, reflecting the average blood glucose level of participants within 1–2 weeks), and cardiorespiratory fitness (CRF). The secondary outcomes included weight, body mass index (BMI), waist circumference, muscular strength, body composition, and physical level (PL). The normal range of waist circumference (<85 cm in men and <80 cm in women) and the standard for BMI (18.5–23.9 kg/m^2^ for normal individuals, 24.0–27.9 kg/m^2^ for overweight, and ≥28 kg/m^2^ for obesity) were estimated according to the Chinese guidelines [13].

Other parameters were calculated as follows: ratio of change (%) = (values following intervention − values prior to intervention)/values prior to intervention × 100%; difference in values = values following intervention − values prior to intervention; negative conversion ratio (%) = number of individuals who became euglycemic following intervention/total number of individuals × 100%; and decrease ratio (%) = number of individuals with indicator decrease following intervention/total number of individuals × 100%. Insulin resistance (IR) was estimated using Homeostasis Model Assessment 2 (HOMA2) software (HOMA Calculator v2.2.3) [14].

CRF submaximal exercise tests were performed using an XC1000 Electronic cycle ergometer with a DSM electrocardiograph and a Tango M2 electro sphygmomanometer. Graded exercise tests (GXT, 3 min per stage, increase to 25 weeks per stage from 0 weeks to 85% HR_max_ predicted by age) were used to estimate oxygen uptake (V̇O_2max_). The following parameters were monitored: HR, blood pressure, and RPE. In addition, an electrocardiogram (ECG) was performed at rest and during the exercise tests, and V̇O_2max_ was estimated by HR at the end of each test stage.

A multiple RM muscular strength test was performed only in group R prior to each training stage to select an appropriate tension of the elastic band according to previous studies [10, 11]. The strength test was performed with the movement and speed/rhythm used in training: 14 to ≥16 RM test for training stage 1, 12–16 RM test for training stage 2, and 8–12 RM test for training stage 3.

The body composition was determined using Tanita MC180 for bioelectrical impedance analysis.

PL was estimated using the PA questionnaire (International Physical Activity Questionnaire–Short Form) [15]. According to the results of the PA questionnaire (IPAQ short questionnaire), a limited number of the enrolled participants were engaged in physical activities other than walking and running. Therefore, pedometers (Omron HJ-151) were used to evaluate daily PA. The pedometer was worn on the right hip constantly for a maximum period of 7 days with the exception of sleeping and bathing. Daily PA was performed excluding the exercise intervention in our program. The daily step number from the pedometers excluding the intervention time was used to indicate the level of daily PA.

The baseline measures before intervention and the follow-up measures following intervention included all the aforementioned measures. The interval between follow-up and the last exercise exceeded 3 days (72 h) and no more than 1 week. Prior to the baseline and follow-up tests, the instructions were sent to the participants.

### Statistical analysis

The normality of the distribution, including the average values of the indicators, was assessed using the Kolmogorov–Smirnov test, and the homogeneity of variance was tested using Levine’s test. All data in this study fitted normal distribution and were expressed as mean ± standard deviation. The change in the average value prior to and following intervention was assessed using the paired-sample *t* test. The one-way analysis of variance was used to analyze differences among groups, and the least significant difference (LSD) (equal variance) and Tamhane’s T2 (equal variances not assumed) tests were used to compare pairwise differences. The chi-square test was used to analyze ratio differences among the groups. The classification variables were expressed as frequency and percentage (%). A simple bivariate correlation was used to assess the relationship between the two variables. When the data complied with the normal distribution pattern, Pearson’s correlation analysis was used. In any other case, Spearman’s rank correlation was used. A correlation index lower than 0.4 indicated a low correlation, whereas an average correlation value of 0–0.7 indicated a moderate correlation. A correlation value higher than 0.7 indicated a high correlation.

SPSS19.0 (IBM, NY, USA) was used to perform statistical analysis. *P* <0.05 and *P* <0.01 indicated significant and extremely significant differences, respectively.

## Results

A total of 80 participants were enrolled in the experiment, of whom 27 were in group A, 26 in group R, and 27 in group C. Nine participants were lost (one in group A, two in group R, and six in group C). The return ratio was 88.8%, with optimal compliance. One participant in group R was excluded from the analysis due to treatment with drugs for paranoia, which influenced blood glucose levels. Finally, 26 participants were included in group A, 23 in group R, and 21 in group C. The baseline participant characteristics are listed in Table 1. No significant differences were found between the groups.

**Table 1:** Baseline subjects’ characteristic

Randomized groups	A	R	C	Total
***N***	26	23	21	70
**Age (years)**	51±9.5	52±8.2	50±6.5	51±8.2
**Height (cm)**	162.1±8.7	163.1±8.0	164.0±6.8	163.0±7.9
**Weight (kg)**	66.4±12.7	65.3±9.7	66.2±9.7	66.0±10.8
**Overweight and obesity (%)**	53.8	56.5	57.1	55.7
**Abnormal waist circumference (%)**	84.6	82.6	76.2	81.4
**Sex/*n***				
M	15	13	11	39
F	11	10	10	31
**Type of IGR/*n***				
IFG	3	4	4	11
IGT	11	12	12	35
IFG+IGT	12	7	5	24

*IFG* Impaired Glucose Tolerance, *IGT* Impaired Fasting Glucose, *IFG+IGT* A type of IGR with both abnormal FPG and abnormal 2hPG following OGTT at the same time

The main results of this study about the changes in FPG, OGTT 2-h PG, GSP, and HOMA2-IR are presented in Table 3. Prior to intervention, no significant differences were noted in FPG (*P* = 0.187, *F* = 1.722), 2-h PG following OGTT (*P* = 0.379, *F* = 0.985), and GSP (*P* = 0.241, *F* = 1.452) between the groups, whereas significant differences (*P* = 0.016, *F* = 4.381) were noted in HOMA2-IR among the three groups examined. Significant differences were observed between groups A and R [*P* = 0.040, 95% CI: −0.591 to −0.015] and between groups R and C [*P* = 0.006, 95% CI: 0.132−0.739] in HOMA2-IR pre-intervention, but no significant differences were found between groups A and C [*P* = 0.373, 95% CI: −0.163 to 0.428]. After the intervention, the parameters FPG (*P* = 0.001, *t* = 3.866) and 2-h PG following OGTT (*P* < 0.001, *t* = 7.387) significantly decreased in group A, whereas the FPG (*P* = 0.019, *t* = 2.524) and 2-h PG following OGTT (*P* < 0.001, *t* = 5.586) also significantly decreased in group R. The decreases in the two groups were significant compared with those in group C [all *P* < 0.001; FPG A vs C 95% CI: −16.389 to −7.709; FPG R vs C 95% CI: −15.154 to −6.225; 2-h PG following OGTT A vs C 95% CI: −36.131 to −20.109; 2-h PG following OGTT A vs C 95% CI: −32.479 to −15.997], whereas the comparison of groups A with B demonstrated no significant differences [FPG *P* = 0.524, 95% CI: −5.594 to 2.875; 2-h PG following OGTT *P* = 0.325, 95% CI: −11.698 to 3.9338]. In group C, FPG (*P* = 0.823, *t* = −0.226) and 2-h PG following OGTT (*P* = 0.022, *t* = 2.473) decreased.

As shown in Table 3, HOMA2-IR in groups A (*P* = 0.026) and R (*P* = 0.001) significantly decreased following intervention, whereas it significantly decreased in group R [*P* = 0.001, 95% CI: −38.021 to −11.050) and group A (*P* = 0.030, 95% CI: −27.679 to −1.461] compared with that in group C, but the differences between group A and R were insignificant [*P* = 0.125, 95% CI: −2.824 to 22.755].

The values of BMI and waist circumference and their ratio of change are also presented in Table 3. Prior to intervention, no significant differences were noted in BMI (*P* = 0.698, *F* = 0.361) and waist circumference (*P* = 0.868, *F* = 0.141) (Table 1). Investigation of the ratio of change of BMI demonstrated that the differences were significant between groups A and C [*P* = 0.009, 95% CI: −4.329 to −0.643] and between groups A and R [*P* = 0.029, 95% CI: −3.807 to −0.211], whereas no significant difference was noted between groups R and C [*P* = 0.617, 95% CI: −2.373 to 1.418]. In addition, the difference was significant between groups A and C [*P* = 0.010, 95% CI: −4.140 to −0.583], whereas no significant difference was noted between groups R and C [*P* = 0.437, 95% CI: −2.547 to 1.113] or between groups A and R [*P* = 0.063, 95% CI: −3.380 to 0.090] with regard to the ratio of change in the waist circumference. The value of FAT% (percentage of body fat) and lean body mass and their ratio of change are shown in Fig. 1B. FAT% in group A following intervention significantly decreased by 5.94 ± 10.69% (*P* = 0.008, *t* = 2.915) on average, whereas the lean body mass also significantly decreased by 0.99 ± 2.17% (*P* = 0.029, *t* = 2.315).

The negative conversion ratio of blood glucose and the decreased ratio of GSP are shown in Table 4. The negative conversion ratio of blood glucose showed significant differences between groups A and C (*P* = 0.003) and groups R and C (*P* = 0.213). The decreased ratio of GSP also showed significant differences between groups R and C (*P* = 0.008).

The correlation analysis examining the ratio of change in BMI, waist circumference, or body composition and the ratio of change in blood glucose levels or IR suggested that only a moderately positive correlation (0.4 < *r* < 0.7) existed between the ratio of BMI change (*r* = 0.498, *P* = 0.010, two-tailed), FAT% (*r* = 0.465, *P* = 0.019, two-tailed), and the ratio of change for FPG in group A. No significant correlations were noted between other indicators.

The data of the step number before and after invention are shown in Table 2. The daily total step number (*P* = 0.959, *F* = 0.042) and the step number above moderate intensity (*P* = 0.669, *F* = 0.404) among the three groups prior to intervention exhibited no significant differences. In addition, no significant differences were noted among the three in the ratio of change in the daily total step number (*P* = 0.197, *F* = 1.667) and the difference in the values of step number above moderate intensity (*P* = 0.434, *F* = 0.846) prior to and following the intervention.

**Table 2:** Average daily total step number and step number above moderate intensity and their ratio of change (%)

Randomized groups	A	R	C
***N***	26	23	17
**Total step number**			
Before intervention (step/day)	9501.9±3407.2	9299.7±3568.0	9183.6±4117.6
After intervention (step/day)	10843.2±3485.7	8897.2±3679.6	9901.7±4397.7
Ratio of change (%)	24.4±51.2	1.6±49.0	9.68±19.2
*P* value	0.092	0.560	0.115
**Above moderate intensity step number**^**a**^			
Before intervention (step/day)	1749.7±1817.2	1662.7±1928.7	2244.1±2791.7
After intervention (step/day)	2656.8±2212.2	2032.3±2697.3	2283.0±2592.7
Difference in values (step/day)	907.2±2335.8	369.6±2541.2	38.9±1393.6
*P* value	0.059	0.493	0.910

Failed to obtain the pedometer data from 4 participants of group C. The data were fitted into a normal distribution in each group

^**a**^ Above moderate intensity step number: the step number of walking speed>4 km/h

## Discussion

The significant decreases noted in FPG and 2-h PG following OGTT in both exercise intervention groups indicated that aerobic and resistance exercise could effectively control and reverse IGR (Tables 3 and 4). This was also supported by the large negative conversion ratio following exercise intervention. Although several studies showed that aerobic exercise improved the blood glucose level in individuals with IGR [7, 16, 17], the influence of resistance exercise on their blood glucose levels was not clear [8, 9]. However, the benefit of resistance exercise for improving blood glucose in patients with T2DM was partially supported by previous evidence [18–20], and no significant differences were noted in the glycemic control group compared with the aerobic exercise group [21, 22]. The effects of blood glucose intervention noted in the present study were in line with the aforementioned findings. However, the average value of GSP in the present study did not significantly decrease compared with the HbA1c outcomes reported in patients with T2DM by previous studies. The possible reason was that the baseline blood glucose level in individuals with IGR was lower than that in patients with DM, whereas the altered range of blood glucose and the amplitude of the average change in blood glucose levels were relatively small (the average ratio of change in FPG and 2-h PG following OGTT was −6.17% to 0.52% and −20.39% to −8.97%, respectively).

**Table 3:** The values of FPG, OGTT 2 h PG, GSP, HOMA2-IR, BMI, and waist circumference and their ratio of change (%)

Randomized groups	A	R	C
***N***	26	23	21
**FPG (mmol/l)**			
Before	6.19±0.6	6.09±0.7	5.86±0.6
After	5.78±0.4**	5.78±0.7*	5.88±0.6
Ratio of change	−6.17±8.5^##^	−4.81±9.3^##^	0.52±6.4
**OGTT 2 h PG (mmol/l)**			
Before	9.05±1.4	9.17±1.7	8.60±1.2
After	7.07±1.1**	7.47±1.1**	7.73±1.4*
Ratio of change	−20.39±17.1^##^	−16.50±15.3^##^	−8.97±19.0
**GSP (μmol/l)**			
Before	197.03±25.5	208.61±49.2	191.75±20.6
After	202.44±29.0	197.75±22.5	203.51±22.5
Ratio of change	3.51±14.0	−1.51±20.8	7.11±14.2
**HOMA2-IR**			
Before	1.97±0.5^&^	2.27±0.6^#^	1.84±0.4
After	1.72±0.3*	1.78±0.3**	1.89±0.3
Ratio of change	−8.34±24.7^#^	−18.31±19.9^##^	6.23±22.0
**BMI (kg/m**^**2**^**)**			
Before	25.1±2.9	24.5±2.7	24.5±2.4
After	24.2±2.6**	24.2±2.3*	24.3±2.2
Ratio of change	−3.1±3.2^#&^	−1.1±2.9	−0.7±3.4
**Waist circumference (cm)**			
Before	89.1±9.0	87.8±8.8	88.6±8.9
After	86.3±8.4**	86.3±7.5*	87.8±8.4
Ratio of change	−3.1±2.7^##^	−1.5±3.8	−0.8±2.5

*P<0.05, **P<0.01, compared with pre-intervention within group; ^#^*P*<0.05, ^##^*P*<0.01, compared with group C; ^&^*P*<0.05, ^&&^*P*<0.01, compared with group R. The data were fitted into a normal distribution in each group

**Table 4:** The negative conversion ratio of blood glucose and the decrease ratio of GSP

Randomized groups	A	R	C	Total
*N*	26	23	21	70
**Negative conversion ratio of blood glucose**				
Negative (*n*)	18	10	5	33
Positive (*n*)	8	13	16	27
Negative conversion ratio (%)	69.2**	43.5	23.8	47.1
**Decrease ratio of GSP**				
Decreased (*n*)	10	15	5	30
Not decreased (*n*)	16	8	16	40
Decreased ratio (%)	38.5	65.2**	23.8	42.9

**P<0.01, compared with group C

This study also demonstrated the different effects caused by aerobic and resistance types of exercise intervention on IGR. The decrease in blood glucose levels was slightly higher in group A than in group R, but the results were not significantly different. This was probably because the increase in PA levels was higher in group A (24.44%) than in group R (1.59%); however, the findings were not affected significantly by daily physical activity other than the intervention, as shown in Table 2. The negative conversion ratio in group A was significantly different from that in group C, whereas no significant differences were found between groups R and C. The possible reason for this outcome was the number of individuals in group C indicating lower FPG and 2-h PG following OGTT prior to intervention. Therefore, a slight decrease resulted in a reversion to euglycemia in participants with blood glucose levels slightly higher than the baseline level. Different findings were noted with regard to GSP in groups A and R, suggesting that resistance exercise decreased GSP in a higher percentage of participants compared with aerobic exercise (significant differences were noted between groups R and C, but not between groups A and C). The large individual differences noted in GSP following resistance exercise might be related to the subjective levels of effort during resistance training, or the individual adaptation and response to resistance exercise. The possible reason explaining a significant decrease in the levels of 2-h PG following OGTT in group C compared with those at pre-intervention might be associated with the effects of health education on diet and PA in group C.

Referring to the effect of exercise intervention on IR, the results (Table 3) showed that both aerobic and resistance exercise improved IR in IGR, and the effect of resistance exercise on IR improvement was superior to that of aerobic exercise. Although IR and insulin secretion dysfunction were previously shown in individuals with IFG and IGT, the physiological basis was not similar. IFG was characterized by liver IR and injury to pancreatic β cells. However, early-stage insulin secretion following oral glucose administration was normal. IGT was mainly related to peripheral IR and, notably, IR in skeletal muscle. In addition, early-stage insulin secretion was attenuated following oral glucose intervention, and the function of pancreatic β cells was normal under basal state conditions [23]. When the decrease in the high secretion state of pancreatic β cells failed to inhibit the increase in blood glucose levels, IGR progressed to DM. In the present study, both aerobic and resistance exercises improved IR in individuals with IGR, which was in line with the findings reported in a previous study [24]. Furthermore, the effect of resistance exercise on IR improvement was superior to that of aerobic exercise, probably due to the small number of individuals with IFG among the enrolled participants (only 3, i.e., 11.5%, Table 1); the value of the 2-h PG following the OGTT of other participants was abnormal. The increase in the levels of 2-h PG following OGTT was closely associated with the IR of skeletal muscle, and resistance exercise might be more effective in improving the IR of skeletal muscle by enhancing muscle metabolism [25] and body composition and increasing muscle mass [26] compared with aerobic exercise. As shown in Fig. 1B, the percentage of fat (FAT%) decreased to a greater extent in group R than in other groups, and an increased lean body mass was noted in group R, although no significant differences were noted in group comparisons. However, in group A, although the FAT% significantly decreased, the lean body mass also significantly decreased. Additional studies are required to accurately define muscle mass and assess the levels of functional indicators so as to explore the relationship between IR and muscle mass and function in IGR.

Moreover, the relationship between exercise intervention effects and obesity was also investigated. Approximately three fourths (Table 1) of the enrolled individuals with IGR indicated increased waist circumference [13], whereas only half of them were overweight and obese. Combining the results of correlation analysis with the results in Table 3 and Fig. 1B, it was found that aerobic exercise lowered the weight and waist circumference significantly in IGR, which was in line with previous findings [7, 17]. Both FAT% and lean body mass decreased significantly in group A probably due to the influence of health education on a diet, which limited the participants’ energy intake (since FAT% and lean body mass also decreased in group C). Although resistance exercise alone failed to decrease weight and waist circumference, resistance exercise caused a slight increase in lean body mass. This might be because health education (increase in high-protein diet intake) prevented the decrease in the lean body mass caused by decreased energy intake [27]. However, the dietary condition still requires further investigation. Although the results suggested that, following the intervention, the decrease in FPG levels in group A was related to the decrease in BMI and FAT%. However, it could not be confirmed that the decrease in FAT% in group A was triggered by exercise. Hence, it was concluded that aerobic exercise decreased blood glucose levels by inducing weight loss in IGR, which was in line with the findings reported in previous studies [7, 17, 28]. Also, the improvement in blood glucose level and IR by resistance exercise in IGR might not be mediated by weight loss and obesity control. Previous studies suggested that resistance exercise decreased blood glucose levels in patients with DM and improved their IR via an increase in muscle mass [19, 27, 29]. However, the present study failed to reinforce the conclusion in participants with IGR, warranting further exploration.

This sample size of the present study could support the main outcome, but the changes in the levels of GSP and body composition, as well as the relationship between different indicators, might not be adequately explained. Therefore, a larger sample size is required to indicate significant differences, and additional studies should be conducted to accurately define muscle mass and functional indicators so as to explore the relationship between IR and muscle mass and their function in the development of IGR. Certain variables, such as diet during the intervention, may affect the significance of the current results. In future studies, an improved control group is required to investigate these variables. Moreover, our study was conducted at a simple research center, resulting in some selection bias among the participants. A multicenter study may resolve this issue.

## Conclusions

In pre-diabetes, both resistance and aerobic exercise can control and reverse IGR. The effect of resistance exercise on GSP and IR improvement may be superior to that of aerobic exercise. Resistance exercise resulted in more individual differences in the control of the mean blood glucose levels compared with aerobic exercise. Aerobic exercise decreased blood glucose levels through weight loss. In contrast, the improvement in blood glucose and IR by resistance exercise in IGR might not be mediated via weight loss and obesity control. This suggested that a better exercise intervention mode might combine resistance exercise with aerobic exercise in pre-diabetes compared with traditional aerobic exercise intervention alone. Moreover, resistance exercise might be more important for certain individuals with severe IR.

## Data Availability

All data generated or analyzed during this study are included in this published article.
